# Acute Biventricular Mechanical Circulatory Support for Cardiogenic Shock

**DOI:** 10.1161/JAHA.117.006670

**Published:** 2017-10-20

**Authors:** Sudeep Kuchibhotla, Michele L. Esposito, Catalina Breton, Robert Pedicini, Andrew Mullin, Ryan O'Kelly, Mark Anderson, Dennis L. Morris, George Batsides, Danny Ramzy, Mark Grise, Duc Thinh Pham, Navin K. Kapur

**Affiliations:** ^1^ Acute Mechanical Circulatory Support Working Group Tufts Medical Center and Tufts University School of Medicine Boston MA; ^2^ The Cardiovascular Center Tufts Medical Center and Tufts University School of Medicine Boston MA; ^3^ Einstein Healthcare Network Philadelphia PA; ^4^ Robert Wood Johnson University Hospital New Brunswick NJ; ^5^ Cedars‐Sinai Medical Center Los Angeles CA; ^6^ Sacred Heart Hospital Pensacola FL

**Keywords:** cardiogenic shock, hemodynamics, mechanical circulatory support, right ventricle‐pulmonary arterial coupling, right ventricular failure, Catheter-Based Coronary and Valvular Interventions, Hemodynamics, Heart Failure

## Abstract

**Background:**

Biventricular failure is associated with high in‐hospital mortality. Limited data regarding the efficacy of biventricular Impella axial flow catheters (BiPella) support for biventricular failure exist. The aim of this study was to explore the clinical utility of percutaneously delivered BiPella as a novel acute mechanical support strategy for patients with cardiogenic shock complicated by biventricular failure.

**Methods and Results:**

We retrospectively analyzed data from 20 patients receiving BiPella for biventricular failure from 5 tertiary‐care hospitals in the United States. Left ventricular support was achieved with an Impella 5.0 (n=8), Impella CP (n=11), or Impella 2.5 (n=1). All patients received the Impella RP for right ventricular (RV) support. BiPella use was recorded in the setting of acute myocardial infarction (n=11), advanced heart failure (n=7), and myocarditis (n=2). Mean flows achieved were 3.4±1.2 and 3.5±0.5 for left ventricular and RV devices, respectively. Total in‐hospital mortality was 50%. No intraprocedural mortality was observed. Major complications included limb ischemia (n=1), hemolysis (n=6), and Thrombolysis in Myocardial Infarction major bleeding (n=7). Compared with nonsurvivors, survivors were younger, had a lower number of inotropes or vasopressors used before BiPella, and were more likely to have both devices implanted simultaneously during the same procedure. Compared with nonsurvivors, survivors had lower pulmonary artery pressures and RV stroke work index before BiPella. Indices of RV afterload were quantified for 14 subjects. Among these patients, nonsurvivors had higher pulmonary vascular resistance (6.8; 95% confidence interval [95% CI], 5.5–8.1 versus 1.9; 95% CI, 0.8–3.0; *P*<0.01), effective pulmonary artery elastance (1129; 95% CI, 876–1383 versus 458; 95% CI, 263–653; *P*<0.01), and lower pulmonary artery compliance (1.5; 95% CI, 0.9–2.1 versus 2.7; 95% CI, 1.8–3.6; *P*<0.05).

**Conclusions:**

This is the largest, retrospective analysis of BiPella for cardiogenic shock. BiPella is feasible, reduces cardiac filling pressures and improves cardiac output across a range of causes for cardiogenic shock. Simultaneous left ventricular and RV device implantation and lower RV afterload may be associated with better outcomes with BiPella. Future prospective studies of BiPella for cardiogenic shock are required.

**Clinical Perspective**

**What Is New?**

- Biventricular support with 2 axial flow Impella catheters (BiPella) improves the hemodynamic profile of patients with cardiogenic shock.
- Compared to staged implantation, simultaneous deployment of left ventricular and right ventricular Impella support devices is associated with improved survival.
- Indices of increased right ventricular afterload in cardiogenic shock are associated with higher in‐hospital mortality among BiPella recipients.

**What Are the Clinical Implications?**

- Nonsurgical deployment of left ventricular and right ventricular Impella catheters for biventricular circulatory support is clinically feasible with no intraprocedural mortality or device failure.
- Careful evaluation of hemodynamic parameters, including indices of right ventricular afterload, is important for guidance of device selection. Algorithms are required to guide optimal device utilization.

## Introduction

**Figure 1:**
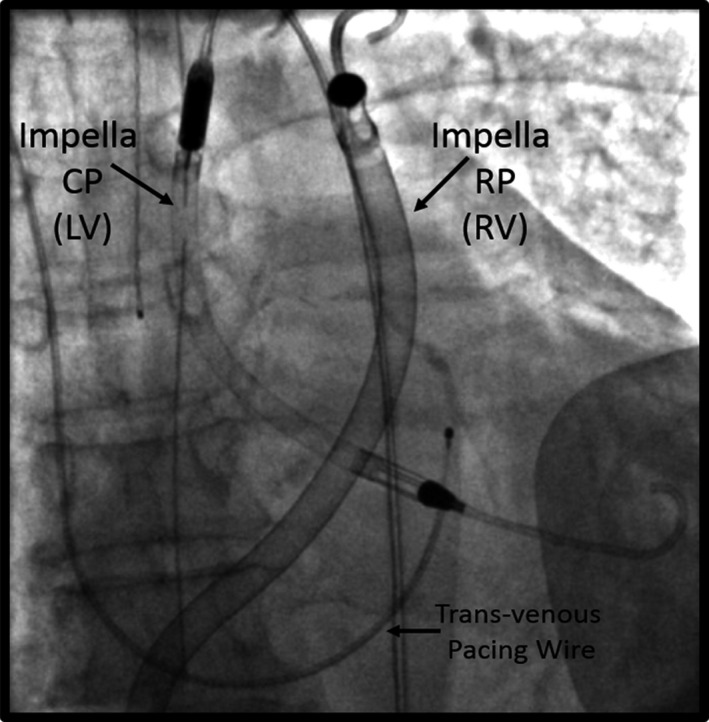
Fluoroscopic image showing biventricular micro‐axial flow Impella catheters for biventricular support (BiPella). LV indicates left ventricular; RV, right ventricular.

Cardiogenic shock (CS) is associated with high in‐hospital mortality.^1^, ^2^ Compared with left ventricular (LV) failure alone, concomitant right ventricular (RV) failure in the setting of CS is associated with higher in‐hospital mortality.^3^, ^4^, ^5^ Biventricular failure (BiVF) can be secondary to acute myocardial infarction, myocarditis, post cardiac surgery, or in the setting of chronic LV failure. Medical therapy for BiVF is limited and includes treatment of the underlying cause of CS, vasopressors, inotropes, and pulmonary vasodilators.^6^ Since 2007, the use of acute mechanical circulatory support (AMCS) devices has grown for CS.^7^, ^8^ AMCS device options include transvalvular microaxial flow catheters (Impella; Abiomed Inc), extracorporeal centrifugal flow pumps for left or right atrial unloading (TandemHeart; TandemLife Inc), or venoarterial extracorporeal membrane oxygenation (VA‐ECMO).^9^, ^10^ Common AMCS strategies for BiVF include VA‐ECMO, biventricular TandemHeart pumps, and various combinations of left‐sided Impella and TandemHeart or VA‐ECMO pumps. Based on results of the Recover Right Trial, the Impella RP is approved for clinical use in the United States as an RV support device.^11^ Since 2013, 3 single‐patient case reports have described the use of 2 microaxial flow Impella catheters for biventricular support (BiPella)^12^, ^13^, ^14^ (Figure 1). The aim of this study was to determine the hemodynamic effect and clinical utility of the BiPella configuration for BiVF in real‐world practice.

## Methods

We retrospectively analyzed the records of 20 patients with BiVF who received BiPella support between 2013 and 2016 from 5 tertiary care institutions. BiPella support was defined as any combination of percutaneously delivered Impella 5.0, Impella CP, or Impella 2.5 for LV support and Impella RP for RV support. The decision to implant BiPella for biventricular support was operator dependent. Patients receiving VA‐ECMO, TandemHeart, or surgically implanted ventricular assist devices (VADs) before BiPella initiation were excluded. All subjects received standard clinical care for BiVF during their index hospitalization. The institutional review board for each participating institution approved this study. Subject informed consent was waived.

Demographic information, hemodynamic and echocardiographic data, laboratory parameters, and outcome data were recorded by investigators at each institution. Implantation sequence and timing as well as flow settings were dictated by operators at each institution without a uniform, predefined protocol or algorithm. To explore the hemodynamic effect of BiPella, pulmonary artery (PA) catheter indices acquired within 24 hours before and after implantation of both LV and RV support devices were compared. In all 20 subjects, both pre‐ and post‐BiPella hemodynamics were available. Cardiac filling pressures, cardiac output (CO) by the Fick method, and established indices of RV function including the right atrial (RA) pressure/pulmonary capillary wedge pressure (PCWP) ratio, the pulmonary artery pulsatility ratio (PA pulse pressure/RA pressure), and RV stroke work index ([mean PA−RA]/stroke volume index). In 14 of 20 subjects, complete hemodynamic data were available for analysis of indices of RV afterload including: pulmonary vascular resistance (PVR; [mean PA−PCWP]/CO); PA compliance (stroke volume/[PA systolic pressure−PA diastolic pressure]); PA elastance ([PA systolic pressure]/stroke volume); and effective PA elastance ([PA systolic pressure−PCWP]/stroke volume). Clinical outcomes including in‐hospital mortality, time to device activation, and device‐associated complications occurring during or within 24 hours of right‐ or left‐sided device implantation or removal were studied. Thrombolysis in Myocardial Infarction major bleeding was defined as either intracranial hemorrhage, clinical signs of hemorrhage associated with a drop in hemoglobin, or fatal bleeding that resulted with in death within 7 days of device implantation.^15^ The presence of device‐associated hemolysis was self‐reported by each institution according to their internal definitions, which were not defined a priori and included either increased serum lactate dehydrogenase or plasma‐free hemoglobin levels, new hemoglobinuria, or new anemia not related to bleeding that required treatment with a blood transfusion.

### Statistical Analysis

Data are expressed as mean±95% confidence interval (95% CI) for continuous variables. Differences between groups and conditions were compared by Student *t* test for continuous variables and Fisher's exact test for categorical variables. Within a survival cohort, pair‐wise *t* tests were used for pre‐ versus postcomparisons whereas data between survival cohorts were analyzed using 2‐sample equal variance *t* tests. All statistical analysis was performed using GraphPad Prism software (GraphPad Software Inc, La Jolla, CA). A *P* value of <0.05 denoted significant difference.

## Results

### Demographics and Implant Characteristics

**Figure 2:**
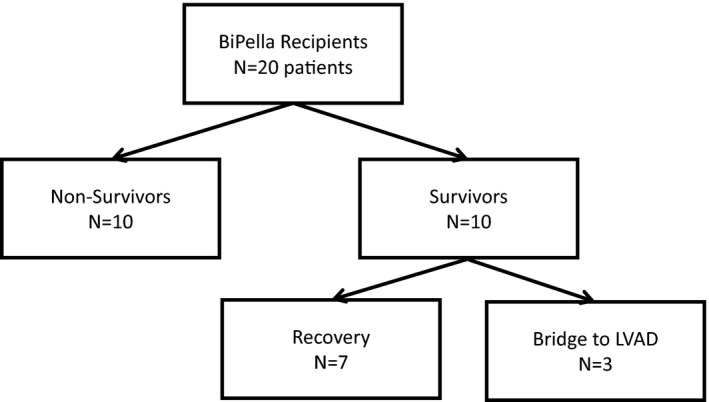
Flow chart of patient outcomes. LVAD, left ventricular assist device.

Baseline characteristics of the total study population stratified by in‐hospital survival are provided in Table 1. In‐hospital mortality among all 20 BiPella recipients was 50%. Of the 10 nonsurvivors, 2 bridged to surgical biventricular VADs (BiVAD) before expiring in the hospital. Of the 10 survivors, 3 bridged to surgical left ventricular assist devices without need for a right ventricular assist device (Figure 2). None of the patients bridged from BiPella to orthotopic heart transplantation or total artificial heart. Compared with nonsurvivors, survivors were more likely to receive simultaneous LV and RV device implantation during the same procedure (90%; 95% confidence interval [95% CI] 70–100 versus 40%; 95% CI, 8–72; *P*=0.02), were younger (52.4; 95% CI, 42.7–62.1 versus 66.8; 95% CI 61.4–72.2; *P*=0.02), and required fewer vasopressors and/or inotropes before device implantation (1.7; 95% CI, 1.1–2.3 versus 2.9; 95% CI, 2.4–3.4; *P*<0.01). Total duration of BiPella support was 5.3 (95% CI, 2.5–7.8) and 4.9 (95% CI, 2.7–7.1) days for nonsurvivors and survivors, respectively (*P*=0.81). No difference in laboratory parameters was observed between groups (Table 2).

**Table 1:** Baseline Demographics

	Total Cohort (n=20)	Nonsurvival (n=10)	Survival (n=10)	*P* Value
Simultaneous implant, %	65	40	90	0.018
Age, y	59.6 (53.3–65.9)	66.8 (61.4–72.2)	52.4 (42.7–62.0)	0.02
Sex (%male)	75	80	70	0.628
Ejection fraction, %	22.8 (17.4–29.3)	21.6 (15.9–27.2)	24.0 (14.7–33.3)	0.674
Level of RV systolic dysfunction	2.1 (1.7–2.6)	2.5 (2.1–2.9)	1.7 (0.9–2.5)	0.106
Severity of tricuspid regurgitation	2.4 (1.8–3.0)	2.1 (1.6–2.7)	2.7 (1.6–3.8)	0.39
Previous myocardial infarction, %	30	40	20	0.355
ICD, %	20	20	20	1
Hypertension, %	70	80	60	0.355
Diabetes mellitus, %	30	50	10	0.054
Atrial fibrillation, %	25	30	20	0.628
Chronic kidney disease, %	25	40	10	0.135
Out‐of‐hospital arrest, %	15	10	20	0.62
Intra‐aortic balloon pump, %	35	30	40	0.66
Pulmonary vasodilators, %	55	60	50	0.91
No. of vasopressors/inotropes	2.3 (1.8–2.8)	2.9 (2.4–3.4)	1.7 (1.1–2.3)	0.005

Range shown is 95% confidence interval of MeanGrade of right ventricular dysfunction (1=mild; 2=moderate; 3=severe). Tricuspid Regurgitation (1=trace; 2=mild; 3=moderate; 4=severe). ICD indicates implantable cardioverter defibrillator; RV, right ventricular.

**Table 2:** Baseline Laboratory Data

	Total Cohort (n=20)	Nonsurvival (n=10)	Survival (n=10)	*P* Value
Sodium, mEq/L	135.7 (133.1–138.3)	136.4 (133.6–139.3)	135.1 (130.8–139.4)	0.627
Potassium, mEq/L	4.4 (4.0–4.7)	4.6 (4.0–5.2)	4.2 (3.8–4.6)	0.285
Chloride, mEq/L	99.4 (95.9–103.0)	99.2 (93.2–105.3)	99.6 (95.3–103.9)	0.92
Bicarbonate, mEq/L	21.8 (19.1–24.4)	21.7 (17.1–26.4)	21.8 (19.2–24.3)	0.992
Blood urea nitrogen, mg/dL	40.8 (31.1–50.5)	50.3 (37.3–63.3)	32.3 (19.8–44.8)	0.066
Creatinine, mg/dL	2.0 (1.5–2.5)	2.4 (1.5–3.2)	1.8 (1.2–2.3)	0.26
Glomerular filtration rate, mL/min per 1.73 m^2^	46.4 (30.9–61.9)	39.2 (15.9–62.5)	51.9 (30.6–73.1)	0.448
Hemoglobin, g/dL	12.4 (10.1–14.6)	11.3 (10.4–12.3)	13.3 (9.1–17.5)	0.411
Hematocrit, %	34.3 (31.3–37.2)	34.7 (31.5–38)	33.8 (28.7–38.9)	0.757
Platelets, K/μL	190.3 (141.5–239.1)	175.9 (119.2–232.6)	203.2 (123.6–282.8)	0.598
Aspartate aminotransferase, IU/L	1673.1 (354.7–2991.5)	1630.8 (0–3632.6)	1710.8 (0–3567.7)	0.955
Alanine aminotransferase, IU/L	1083.0 (298.1–1867.9)	937.4 (13.8–1860.9)	1212.4 (0–2495.8)	0.744
Alkaline phosphatase, IU/L	89.0 (78.8–99.2)	88.3 (69.3–107.2)	89.8 (80.3–99.2)	0.892
Total bilirubin, mg/dL	2.0 (1.3–2.7)	2.6 (1.4–3.7)	1.5 (0.9–2.1)	0.125
Lactate dehydrogenase, IU/L	1173.1 (262.3–2084.0)	1064.7 (0–2329.2)	1255.5 (0–2698.4)	0.86
Lactate, mEq/L	3.4 (2.6–4.2)	3.6 (2.5–4.6)	3.2 (1.8–4.6)	0.675
International normalized ratio	1.8 (1.4–2.1)	1.9 (1.3–2.5)	1.6 (1.3–1.9)	0.394
Arterial pH	7.4 (7.3–7.4)	7.3 (7.2–7.4)	7.4 (7.4–7.5)	0.102

Indications for BiPella included acute myocardial infarction (55%; n=11), acutely decompensated heart failure (35%; n=7), and myocarditis (10%; n=2). All acute myocardial infarction patients underwent emergent and successful percutaneous coronary revascularization. Left‐sided support was provided with an Impella 5.0 (n=8), Impella CP (n=11), or Impella 2.5 (n=1). Impella 5.0 was implanted by the axillary route in 7 of 8 patients. All other patients received Impella 5.0, CP, or 2.5 devices by a femoral artery. All patients received an Impella RP by a femoral vein for RV support. Mean flows achieved were 3.4±1.2 and 3.5±0.5 for LV and RV devices, respectively. No intraprocedural mortality was observed with any device implantation. No device failure requiring explant was reported. Major complications included limb ischemia (5%; n=1), hemolysis (30%; n=6), and Thrombolysis in Myocardial Infarction major bleeding (35%; n=7).

### Hemodynamic Variables and Clinical Outcomes

All 20 patients had a cardiac index <2.2 despite inotropic or vasopressor support (2.3; 95% CI, 1.8–2.8 L/min per m^2^), elevated RA pressure (21; 95% CI, 18.2–22.9 mm Hg), elevated PCWP (25; 95% CI, 22.0–27.1 mm Hg), high RA/PCWP ratios (0.9; 95% CI, 0.8–1.0), low pulmonary artery pulsatility ratios (1.1; 95% CI, 0.8–1.4), and low LV cardiac power output (0.53; 95% CI, 0.43–0.63 W). Compared with survivors, nonsurvivors had higher baseline PA systolic pressures (55; 95% CI, 48–63 versus 44; 95% CI, 37–50; *P*=0.04), PA diastolic pressures (32; 95% CI, 29–34 versus 26; 95% CI, 22–30; *P*=0.03), mean PA pressures (40; 95% CI, 36–44 versus 32; 95% CI, 28–36; *P*=0.01), and RV stroke work index (1122; 95% CI, 831–1414 versus 576; 95% CI, 375–779; *P*=0.007).

**Figure 3:**
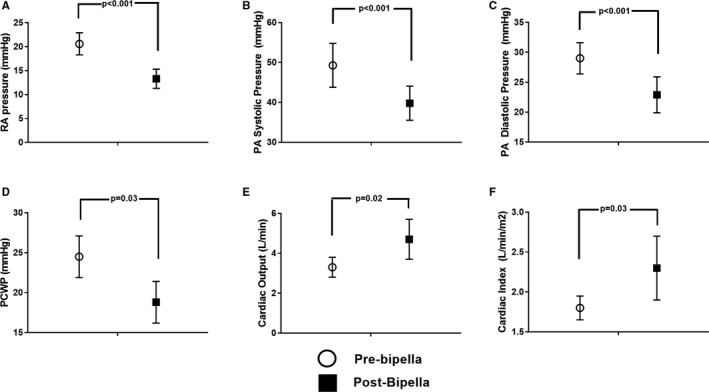
Pre‐ versus posthemodynamic effects of BiPella support among the total cohort: A) Right atrial (RA) pressure. B) Pulmonary artery (PA) systolic pressure. C) PA diastolic pressure. D) Pulmonary capillary wedge pressure (PCWP). E) Cardiac output. F) Cardiac Index. BiPella reduced right atrial (RA) pressure, pulmonary artery (PA) systolic and diastolic pressures, and pulmonary capillary wedge pressure (PCWP) and increased both cardiac output and cardiac index.

Among all 20 patients, Bipella reduced cardiac filling pressures (Table 3). Bipella increased CO (3.3; 95% CI, 2.8–3.8 versus 4.7; 95% CI, 3.7–5.7 L/min; *P*=0.02) and index (1.8; 95% CI, 1.6–1.9 versus 2.3; 95% CI, 1.9–2.7 L/min per m^2^; *P*=0.03; Figure 3). Among nonsurvivors, BiPella reduced RA pressure (20; 95% CI, 17–24 versus 12; 95% CI, 10–15 mm Hg; *P*<0.01), PA systolic pressures (55; 95% CI, 48–63 versus 40; 95% CI, 35–45 mm Hg; *P*<0.01), PA diastolic pressures (32; 95% CI, 29–34 versus 23; 95% CI, 19–26 mm Hg; *P*<0.01), and mean PA pressures (40; 95% CI, 36–44 versus 28; 95% CI, 25–32 mm Hg; *P*<0.01). CO (3.0; 95% CI, 2.3–3.6 versus 4.6; 95% CI, 3.1–6.1 L/min; *P*=0.07) and index (1.7; 95% CI, 1.4–1.9 versus 2.3; 95% CI, 1.6–2.9 L/min per m^2^; *P*=0.08) were also increased, but these values were not statistically significant. Among survivors, BiPella reduced RA pressure (21; 95% CI, 18–24 versus 15; 95% CI, 11–19 mm Hg; *P*=0.02), PA diastolic pressures (26; 95% CI, 22–30 versus 24; 95% CI, 17–30 mm Hg; *P*=0.04), PCWP (23; 95% CI, 20–26 versus 19; 95% CI, 12–27 mm Hg; *P*=0.02), and increased both CO (3.7; 95% CI, 2.9–4.4 versus 5.0; 95% CI, 4.4–5.7 L/min; *P*<0.03) with a trend toward increased cardiac index (1.8; 95% CI, 1.6–2.0 versus 2.2; 95% CI, 1.7–2.7 L/min per m^2^; *P*=0.07).

**Table 3:** Hemodynamics in BiPella Recipients

	Survivors		Nonsurvivors	
	Pre‐BiPella	Post‐BiPella	Pre‐BiPella	Post‐BiPella
RA pressure, mm Hg	20.8 (17.5–24.1)	15.2 (11.4–19.0)^a^	20.3 (16.9–23.7)	12.3 (10.0–14.6)^a^
PA systolic pressure, mm Hg	43.5 (37.0–50.0)	39.3 (30.6–47.9)	55.1 (47.5–62.7)^b^	40.1 (35.4–44.8)^a^
PA diastolic pressure, mm Hg	26.1 (22.3–29.9)	23.5 (17.3–29.7)^a^	31.8 (29.2–34.4)^b^	22.6 (19.3–25.9)^a^
Mean PA pressure, mm Hg	32.2 (28.0–36.4)	29.2 (22.3–36.0)	40.1 (36.4–43.8)^b^	28.4 (25.0–31.8)^a^
RA:PCWP ratio	0.9 (0.7–1.1)	0.8 (0.3–1.2)	0.9 (0.7–1.0)	0.6 (0.5–0.7)
RVSWI, mm Hg·L/m^2^	576 (375.2–778.5)	522.2 (162.3–882.0)	1122 (831.4–1413.6)^b^	729.1 (464.8–993.4)
Pulmonary artery pulsatility index (PAPi)	0.9 (0.5–1.3)	1.0 (0.7–1.4)	1.2 (0.8–1.7)	1.5 (1.1–2.0)
PCWP, mm Hg	23.1 (20.2–26.1)	19.3 (12.1–26.5)^a^	26.1 (21.8–30.5)	18.4 (16.6–20.2)
Cardiac output, L/min	3.7 (2.9–4.4)	5.0 (4.4–5.7)^a^	3.0 (2.3–3.6)	4.6 (3.1–6.1)
Cardiac index, L/min per m^2^	1.8 (1.6–2.0)	2.2 (1.7–2.7)	1.7 (1.4–1.9)	2.3 (1.6–2.9)
Systemic vascular resistance (SVR), mm Hg·min/mL	1299 (924.0–1675.5)	881 (713–1050)	1381 (990.4–1772.8)	1118.2 (552.8–1683.7)

PA indicates pulmonary artery; PCWP, pulmonary capillary wedge pressure; RA, right atrial; RVSWI, right ventricular stroke work index.

a *P*<0.05 for pre‐ vs posthemodynamics within outcome cohorts.

b *P*<0.05 for survivors vs nonsurvivors prehemodynamics.

There are no statistically significant differences for survivors vs nonsurvivors posthemodynamics.

### Analysis of RV Afterload Indices

**Figure 4:**
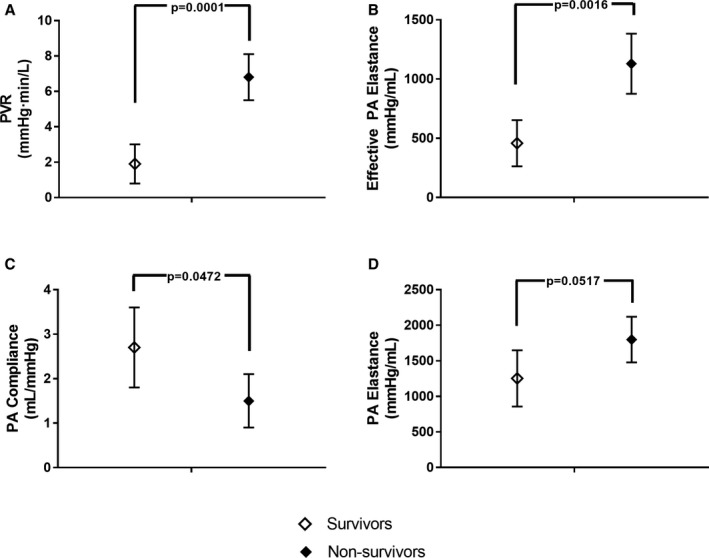
Baseline indices of right ventricular afterload before BiPella support among survivors and nonsurvivors: A) Pulmonary vascular resistance (PVR). B) Effective pulmonary artery (PA) elastance. C) PA compliance. D) PA elastance. Survivors had lower pulmonary vascular resistance (PVR), lower effective pulmonary artery (PA) Elastance, and higher PA compliance compared with nonsurvivors.

**Figure 5:**
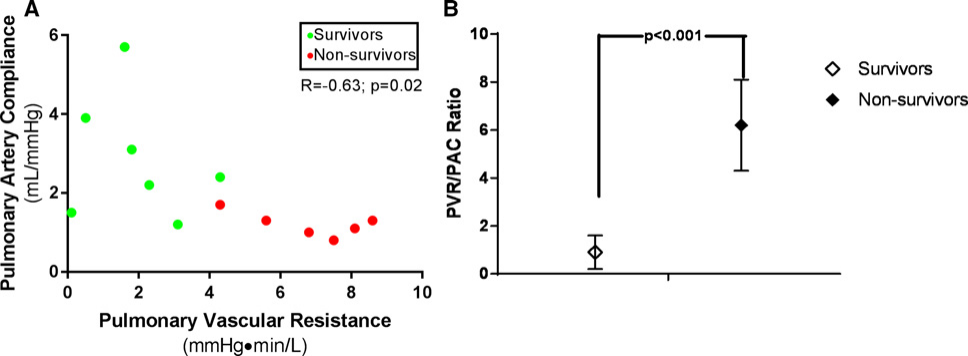
Baseline pulmonary vascular resistance (PVR) and pulmonary artery (PA) compliance among BiPella patients. A, A regression plot shows that PVR inversely correlates with PA compliance. B, The ratio of PVR:PA compliance (PAC) was lower among survivors compared with nonsurvivors.

Based on these data, in‐hospital mortality appeared to be associated with pulmonary hypertension before BiPella implantation. To explore this further, we next analyzed hemodynamic determinants of RV afterload. Compared with survivors, nonsurvivors had higher PVR (6.8; 95% CI, 5.5–8.1 versus 1.9; 95% CI, 0.8–3.0; *P*<0.001), PA elastance (1799; 95% CI, 1479–2120 versus 1253; 95% CI, 857–1649; *P*=0.05), effective PA elastance (1129; 95% CI, 876–1383 versus 458; 95% CI, 263–653; *P*<0.01), and lower PA compliance (1.5; 95% CI, 0.9–2.1 versus 2.7; 95% CI, 1.8–3.6; *P*<0.05; Figure 4). PVR and PA compliance were inversely related (*R*=−0.6; *P*<0.05) and the PVR/PA compliance ratio was higher among nonsurvivors (6.2; 95% CI, 4.2–8.2 versus 1.1; 95% CI, 0.2–1.7; nonsurvivors versus survivors; *P*<0.001; Figure 5).

## Discussion

This report is the largest, retrospective analysis of patients receiving 2 percutaneously delivered microaxial flow Impella catheters for LV and RV support in the setting of CS complicated by biventricular failure. The central finding of this report is that BiPella is clinically feasible and associated with reduced cardiac filling pressures and improved CO across a range of causes for CS. Overall in‐hospital mortality remained high for this critically ill population with severely depressed LV function, massively elevated biventricular filling pressures, reduced RV function, evidence of multiorgan impairment, and elevated lactate levels. Demographic characteristics favoring survival among Bipella recipients for CS included lower age, reduced vasopressor and inotrope requirement before BiPella support, and implantation of LV and RV devices during the same procedure. A closer look at the hemodynamic condition of patients in this study identified that survivors had lower PA pressures compared with nonsurvivors. Based on this observation, we studied indices of RV afterload and identified that high PVR, high effective PA elastance, and lower PA compliance were associated with in‐hospital mortality. These findings identify, for the first time, that BiPella support is feasible, improves hemodynamic conditions in CS, and may be more effective in patients with low PA pressures and reduced RV afterload.

The use of surgical VADs has increased exponentially with over 13 000 implants between 2006 and 2014.^16^ The use of BiVADs accounts for only 5% of all implants for primary LV failure; however, BiVADs are associated with higher short‐ and long‐term mortality compared with isolated left ventricular assist device support.^16^ Multiple past studies have identified risk factors for requiring BiVAD support among patients referred for left ventricular assist device implantation, which include higher vasopressor or inotropic requirements, low RV stroke work, high PVR, and laboratory parameters consistent with impaired hepatic or renal function.^17^, ^18^, ^19^, ^20^, ^21^ In addition, past studies have identified that elective BiVAD implantation during the same procedure is associated with improved outcomes compared with delayed initiation of RV support after left ventricular assist device implantation.^22^, ^23^ No past studies have explored these observations in the setting of percutaneously delivered acute mechanical circulatory support pumps for biventricular failure. We have shown that 90% of patients who survived received a simultaneous implant of their AMCS devices. Our results, in conjunction with the established data from the surgical community showing improved survival with simultaneous biventricular support, imply that simultaneous implant of AMCS devices might also be associated with improved survival with percutaneous support.

Our findings have several major implications for the field of mechanical circulatory support. First, we identified that nonsurgical, rapid deployment of LV and RV Impella catheters for biventricular circulatory support is clinically feasible across multiple centers without intraprocedural mortality or device failure. This represents a significant advance in our ability to provide hemodynamic support for patients with cardiogenic shock. Past biventricular support approaches include use of extracorporeal centrifugal flow pumps and include the TandemHeart device or VA‐ECMO.^24^, ^25^ The TandemHeart device requires a trans‐septal puncture to deliver an inflow cannula into the left atrium for LV support. As a result, the TandemHeart BiVAD requires three 21‐Fr venous cannulas (trans‐septal, RA, and PA cannulas) and a 15‐ to 19‐Fr arterial cannula. This approach is technically challenging to accomplish and may be associated with a high rate of bleeding.^26^ VA‐ECMO alone fails to provide biventricular support and often requires concomitant use of a mechanism to “vent” or reduce LV pressures, which may include an Impella LV catheter, intra‐aortic balloon pump, or drainage cannula in the left atrium.^27^, ^28^, ^29^ BiPella utilizes 2 axial flow pumps mounted on 11‐Fr (RV) and 9‐Fr (LV) catheters that are deployed by a femoral venous and arterial access site, respectively.^12^, ^13^, ^14^ We identified a relatively low rate of complications, including hemolysis and Thrombolysis in Myocardial Infarction major bleeding and a single episode of limb ischemia in a patient receiving Impella CP support by percutaneous puncture of the left axillary artery. An unresolved limitation of all 3 contemporary percutaneous biventricular support approaches is the requirement for femoral vascular access, which restricts patient mobility. The Impella LV catheters can be deployed by the axillary artery; however, the Impella RP device currently requires femoral venous access.

Second, consistent with reports from the surgical VAD literature, we identified that higher preprocedural vasopressor and/or inotropic requirements and increased lactate levels are associated with higher mortality. Both of these indices may be considered markers of a more‐prolonged duration of cardiogenic shock leading to metabolic failure. At this stage, acute mechanical circulatory support may be insufficient to improve survival. These findings highlight the importance of early identification and referral for acute mechanical circulatory support in patients with cardiogenic shock.

Third, we identified that, among BiPella recipients, in‐hospital mortality is associated with elevated PA pressures and that, compared with survivors, nonsurvivors also had a higher RV stroke work index before BiPella implantation. These observations suggest that these patients may not have had biventricular failure, but rather had CS attributed to LV failure with secondary pulmonary hypertension and subsequent biventricular congestion. The nonsurvivor cohort may therefore not have benefited from implantation of a biventricular support device. Recent data suggest that elevated left‐sided filling pressures may serve as an “amplifier” of RV afterload by reducing PA compliance and increasing PVR.^30^ Our analysis identified that indices of increased RV afterload, including PVR, PA compliance, and both PA elastance and effective PA elastance (corrected for PCWP), are associated with poor survival.^31^, ^32^ We also observed that reduced PA compliance inversely correlated with PVR and furthermore that the ratio of these 2 variables correlates with survival among Bipella recipients. These observations suggest that more than 1 component of RV afterload is associated with clinical outcomes among Bipella recipients and that, with further study, hemodynamic indices with prognostic implications for patients with biventricular failure may be developed. One explanation for this observation is that elevated RV afterload may be an indicator of disease severity and chronicity, suggesting that BiPella support may not improve survival. Furthermore, given that rotary flow pumps are sensitive to afterload, high RV afterload may be associated with lower flow through the RV support system.^33^ Finally, increased PVR may be fixed, as opposed to reversible, thereby serving as an obstruction to antegrade flow from an RV support system and limiting LV preload. In cases with severely elevated PVR, reducing RV preload with VA‐ECMO and a concomitant LV vent may be a better alternative to BiPella. Collectively, our findings suggest that close attention to pulmonary vascular load indices may help identify potential candidates for BiPella therapy.

Limitations of our study include the small number of patients studied, which limited our ability to perform subgroup analyses. Furthermore, the retrospective nature of the analysis limited our ability to rigorously identify predictors of outcomes associated with BiPella therapy. Additionally, this was a multicenter study in which indications for BiPella implantation varied between institutions. The combinations of left‐ and right‐sided support devices also differed between patients and may have had effects on outcomes. Future studies with larger cohorts of patients are required.

In conclusion, use of acute mechanical circulatory support devices for cardiogenic shock is growing. The ability to now provide biventricular circulatory support using 2 microaxial flow Impella catheters opens many new possibilities for the treatment of CS and advanced heart failure. Furthermore, the availability of these powerful tools mandates us all to become more familiar with advanced hemodynamics, especially around biventricular interactions and the right ventricular‐pulmonary arterial axis. Findings from this analysis may support the development of future treatment algorithms and prospective studies evaluating the use of BiPella in patients with acute myocardial infarction, acutely decompensated heart failure, pulmonary hypertension, or as a bridge to durable VAD therapy.

## Disclosures

Dr Kapur receives research funding from Maquet Inc, Cardiac Assist Inc, Abiomed Inc, St Jude Inc, and Boston Scientific Inc and speaker/consultant honoraria from Maquet Inc, Abiomed Inc, St. Jude Inc, and Heartware Inc.

## References

[1] Menees DS , Peterson ED , Wang Y , Curtis JP , Messenger JC , Rumsfeld JS , Gurm HS . Door‐to‐balloon time and mortality among patients undergoing primary PCI. N Engl J Med. 2013;369:901–909. 24004117 10.1056/NEJMoa1208200

[2] McNamara RL , Kennedy KF , Cohen DJ , Diercks DB , Moscucci M , Ramee S , Wang TY , Connolly T , Spertus JA . Predicting in‐hospital mortality in patients with acute myocardial infarction. J Am Coll Cardiol. 2016;68:626–635. 27491907 10.1016/j.jacc.2016.05.049

[3] Mehta SR , Eikelboom JW , Natarajan MK , Diaz R , Yi C , Gibbons RJ , Yusuf S . Impact of right ventricular involvement on mortality and morbidity in patients with inferior myocardial infarction. J Am Coll Cardiol. 2001;37:37–43. 11153770 10.1016/s0735-1097(00)01089-5

[4] Voelkel NF , Quaife RA , Leinwand LA . Right ventricular function and failure: report of a National Heart, Lung, and Blood Institute Working Group on Cellular and Molecular Mechanisms of Right Heart Failure. Circulation. 2006;114:1883–1891. 17060398 10.1161/CIRCULATIONAHA.106.632208

[5] Zehender M , Kasper W , Kauder E . Right ventricular infarction as an independent predictor of prognosis after acute inferior myocardial infarction. N Engl J Med. 1993;328:981–988. 8450875 10.1056/NEJM199304083281401

[6] Greyson CR . Pathophysiology of right ventricular failure. Crit Care Med. 2008;36(1 Suppl):S57–S65. 18158479 10.1097/01.CCM.0000296265.52518.70

[7] Wayangankar SA , Bangalore S , McCoy LA , Jneid H , Latif F , Karrowni W , Charitakis K , Feldman DN , Dakik HA , Mauri L , Peterson ED , Messenger J , Roe M , Mukherjee D , Klein A . Temporal trends and outcomes of patients undergoing percutaneous coronary interventions for cardiogenic shock in the setting of acute myocardial infarction: a report from the CathPCI Registry. JACC Cardiovasc Interv. 2016;9:341–351. 26803418 10.1016/j.jcin.2015.10.039

[8] Stretch R , Sauer CM , Yuh DD , Bonde P . National trends in the utilization of short‐term mechanical circulatory support: incidence, outcomes, and cost analysis. J Am Coll Cardiol. 2014;64:1407–1415. 25277608 10.1016/j.jacc.2014.07.958

[9] Morine KJ , Kapur NK . Percutaneous mechanical circulatory support for cardiogenic shock. Curr Treat Options Cardiovasc Med. 2016;18:6. 26758053 10.1007/s11936-015-0426-6

[10] Burkhoff D , Sayer G , Doshi D , Uriel N . Hemodynamics of mechanical circulatory support. J Am Coll Cardiol. 2015;66:2663–2674. 26670067 10.1016/j.jacc.2015.10.017

[11] Anderson MB , Goldstein J , Milano C , Morris LD , Kormos RL , Bhama J , Kapur NK , Bansal A , Garcia J , Baker JN , Silvestry S , Holman WL , Douglas PS , O'Neill W . Benefits of a novel percutaneous ventricular assist device for right heart failure: the prospective RECOVER RIGHT study of the Impella RP device. J Heart Lung Transplant. 2015;34:1549–1560. 26681124 10.1016/j.healun.2015.08.018

[12] Hunziker P , Hunziker L . Percutaneous biventricular cardiac assist device in cardiogenic shock. Eur Heart J. 2013;34:1620. 23594594 10.1093/eurheartj/eht020

[13] Kapur NK , Jumean M , Ghuloom A , Aghili N , Vassallo C , Kiernan MS , DeNofrio D , Pham DT . First successful use of 2 axial flow catheters for percutaneous biventricular circulatory support as a bridge to a durable left ventricular assist device. Circ Heart Fail. 2015;8:1006–1008. 26374919 10.1161/CIRCHEARTFAILURE.115.002374

[14] Aghili N , Bader Y , Vest AR , Kiernan MS , Kimmelstiel C , DeNofrio D , Kapur NK . Biventricular circulatory support using 2 axial flow catheters for cardiogenic shock without the need for surgical vascular access. Circ Cardiovasc Interv. 2016;9:e003636. 27188188 10.1161/CIRCINTERVENTIONS.116.003636

[15] Mehta SK , Frutkin AD , Lindsey JB , House JA , Spertus JA , Rao SV , Ou FS , Roe MT , Peterson ED , Marso SP . Bleeding in patients undergoing percutaneous coronary intervention: the development of a clinical risk algorithm from the National Cardiovascular Data Registry. Circ Cardiovasc Interv. 2009;2:222–229. 20031719 10.1161/CIRCINTERVENTIONS.108.846741

[16] Kirklin JK , Naftel DC , Pagani FD , Kormos RL , Stevenson LW , Blume ED , Myers SL , Miller MA , Baldwin JT , Young JB . Seventh INTERMACS annual report: 15,000 patients and counting. J Heart Lung Transplant. 2015;34:1495–1504. 26520247 10.1016/j.healun.2015.10.003

[17] Arigiriou M , Kolokotron SM , Sakellaridis T , Argiriou O , Charitos C , Zarogoulidis P , Katsikogiannis N , Kougioumtzi I , Machairiotis N , Tsiouda T , Tsakiridis K , Zarogoulidis K . Right heart failure post left ventricular assist device implantation. J Thorac Dis. 2014;6(Suppl 1):S52–S59. 24672699 10.3978/j.issn.2072-1439.2013.10.26 PMC3966152

[18] Pettinari M , Jacobs S , Rega F , Verbelen T , Droogne W , Meyns B . Are right ventricular risk scores useful? Eur J Cardiothorac Surg. 2012;42:621–626. 22518041 10.1093/ejcts/ezs104

[19] Fitzpatrick JR III , Frederick JR , Hsu VM , Kozin ED , O'Hara ML , Howell E , Dougherty D , McCormick RC , Laporte CA , Cohen JE , Southerland KW , Howard JL , Jessup ML , Morris RJ , Acker MA , Woo YJ . Risk score derived from pre‐operative data analysis predicts the need for biventricular mechanical circulatory support. J Heart Lung Transplant. 2008;27:1286–1292. 19059108 10.1016/j.healun.2008.09.006 PMC3235680

[20] Drakos SG , Janicki L , Horne BD , Kfoury AG , Reid BB , Clayson S , Horton K , Haddad F , Li DY , Renlund DG , Fisher PW . Risk factors predictive of right ventricular failure after left ventricular assist device implantation. Am J Cardiol. 2010;105:1030–1035. 20346326 10.1016/j.amjcard.2009.11.026

[21] Alba AC , Rao V , Ivanov J , Ross HJ , Delgado DH . Usefulness of the INTERMACS scale to predict outcomes after mechanical assist device implantation. J Heart Lung Transplant. 2009;28:827–833. 19632580 10.1016/j.healun.2009.04.033

[22] Takeda K , Naka Y , Yang JA , Uriel N , Colombo PC , Jorde UP , Takayama H . Outcome of unplanned right ventricular assist device support for severe right heart failure after implantable left ventricular assist device insertion. J Heart Lung Transplant. 2014;33:141–148. 23932442 10.1016/j.healun.2013.06.025

[23] Fitzpatrick JR III , Frederick JR , Hiesinger W , Hsu VM , McCormick RC , Kozin ED , Laporte CM , O'Hara ML , Howell E , Dougherty D , Cohen JE , Southerland KW , Howard JL , Paulson EC , Acker MA , Morris RJ , Woo YJ . Early planned institution of biventricular mechanical circulatory support results in improved outcomes compared with delayed conversion of a left ventricular assist device to a biventricular assist device. J Thorac Cardiovasc Surg. 2009;137:971–977. 19327526 10.1016/j.jtcvs.2008.09.021 PMC3232461

[24] Kar B , Gregoric ID , Basra SS , Idelchik GM , Loyalka P . The percutaneous ventricular assist device in severe refractory cardiogenic shock. J Am Coll Cardiol. 2011;57:688–696. 20950980 10.1016/j.jacc.2010.08.613

[25] Pavlushkov E , Berman M , Valchanov K . Cannulation techniques for extracorporeal life support. Ann Transl Med. 2017;5:70. 28275615 10.21037/atm.2016.11.47 PMC5337209

[26] Kapur NK , Paruchuri V , Jagannathan A , Steinberg D , Chakrabarti AK , Pinto D , Aghili N , Najjar S , Finley J , Orr NM , Tempelhof M , Mudd JO , Kiernan MS , Pham DT , DeNofrio D . Mechanical circulatory support for right ventricular failure. JACC Heart Fail. 2013;1:127–134. 24621838 10.1016/j.jchf.2013.01.007

[27] Koeckert MS , Jorde UP , Naka Y , Moses JW , Takayama H . Impella LP 2.5 for left ventricular unloading during venoarterial extracorporeal membrane oxygenation support. J Card Surg. 2011;26:666–668. 22122379 10.1111/j.1540-8191.2011.01338.x

[28] Chaparro SV , Badheka A , Marzouka GR , Tanawuttiwat T , Ahmed F , Sacher V , Pham SM . Combined use of Impella left ventricular assist device and extracorporeal membrane oxygenation as a bridge to recovery in fulminant myocarditis. ASAIO J. 2012;58:285–287. 22395121 10.1097/MAT.0b013e31824b1f70

[29] Jumean M , Pham DT , Kapur NK . Percutaneous bi‐atrial extracorporeal membrane oxygenation for acute circulatory support in advanced heart failure. Catheter Cardiovasc Interv. 2015;85:1097–1099. 25529821 10.1002/ccd.25791

[30] Tedford RJ , Hassoun PM , Mathai SC , Girgis RE , Russell SD , Thiemann DR , Cingolani OH , Mudd JO , Borlaug BA , Redfield MM , Lederer DJ , Kass DA . Pulmonary capillary wedge pressure augments right ventricular pulsatile loading. Circulation. 2012;125:289–297. 22131357 10.1161/CIRCULATIONAHA.111.051540 PMC3264431

[31] Amin A , Taghavi S , Esmaeilzadeh M , Bakhshandeh H , Naderi N , Maleki M . Pulmonary arterial elastance for estimating right ventricular afterload in systolic heart failure. Congest Heart Fail. 2011;17:288–293. 22103920 10.1111/j.1751-7133.2011.00222.x

[32] Morimont P , Lambermont B , Ghuysen A , Gerard P , Kolh P , Lancellotti P , Tchana‐Sato V , Desaive T , D'Orio V . Effective arterial elastance as an index of pulmonary vascular load. Am J Physiol Heart Circ Physiol. 2008;294:H2736–H2742. 18424634 10.1152/ajpheart.00796.2007

[33] Moazami N , Fukamachi K , Kobayashi M , Smedira NG , Hoercher KJ , Massiello A , Lee S , Horvath DJ , Starling RC . Axial and centrifugal continuous‐flow rotary pumps: a translation from pump mechanics to clinical practice. J Heart Lung Transplant. 2013;32:1–11. 23260699 10.1016/j.healun.2012.10.001

